# Endosymbionts of Metazoans Dwelling in the PACManus Hydrothermal Vent: Diversity and Potential Adaptive Features Revealed by Genome Analysis

**DOI:** 10.1128/AEM.00815-20

**Published:** 2020-10-15

**Authors:** Leilei Li, Minxiao Wang, Lifeng Li, Zengfeng Du, Yan Sun, Xiaocheng Wang, Xin Zhang, Chaolun Li

**Affiliations:** a Key Laboratory of Marine Ecology and Environmental Sciences, Institute of Oceanology, Chinese Academy of Sciences, Qingdao, China; b CAS Center for Ocean Mega-Science, Chinese Academy of Sciences, Qingdao, China; c Laboratory for Marine Ecology and Environmental Sciences, Qingdao National Laboratory for Marine Science and Technology, Qingdao, China; d Beijing GensKey Technology Co., Ltd., Beijing, China; e Key Laboratory of Marine Geology and Environment, Institute of Oceanology, Chinese Academy of Sciences, Qingdao, China; f Deep-Sea Research Center, Institute of Oceanology, Chinese Academy of Sciences, Qingdao, China; g Laboratory of Marine Geology, Qingdao National Laboratory for Marine Science and Technology, Qingdao, China; h National Marine Environmental Monitoring Center, Dalian, China; i University of Chinese Academy of Sciences, Beijing, China; Kyoto University

**Keywords:** endosymbionts, genome analysis, hydrothermal vents, environmental geochemical gradient

## Abstract

Deep-sea hydrothermal vent communities are dominated by invertebrates, namely, bathymodiolin mussels, siboglinid tubeworms, and provannid snails. Symbiosis is considered key to successful colonization by these sedentary species in such extreme environments. In the PACManus vent fields, snails, tubeworms, and mussels each colonized a niche with distinct geochemical characteristics. To better understand the metabolic potentials and genomic features contributing to host-environment adaptation, we compared the genomes of the symbionts of Bathymodiolus manusensis, Arcovestia ivanovi, and Alviniconcha boucheti sampled at PACManus, and we discuss their environmentally adaptive features. We found that B. manusensis and A. ivanovi are colonized by *Gammaproteobacteria* from distinct clades, whereas endosymbionts of B. manusensis feature high intraspecific heterogeneity with differing metabolic potentials. A. boucheti harbored three novel *Epsilonproteobacteria* symbionts, suggesting potential species-level diversity of snail symbionts. Genome comparisons revealed that the relative abundance of gene families related to low-pH homeostasis, metal resistance, oxidative stress resistance, environmental sensing/responses, and chemotaxis and motility was the highest in A. ivanovi’s symbiont, followed by symbionts of the vent-mouth-dwelling snail A. boucheti, and was relatively low in the symbiont of the vent-periphery-dwelling mussel B. manusensis, which is consistent with their environmental adaptations and host-symbiont interactions. Gene families classified as encoding host interaction/attachment, virulence factors/toxins, and eukaryotic-like proteins were most abundant in symbionts of mussels and least abundant in those of snails, indicating that these symbionts may differ in their host colonization strategies. Comparison of *Epsilonproteobacteria* symbionts to nonsymbionts demonstrated that the expanded gene families in symbionts were related to vitamin B_12_ synthesis, toxin-antitoxin systems, methylation, and lipopolysaccharide biosynthesis, suggesting that these are vital to symbiont establishment and development in *Epsilonproteobacteria*.

**IMPORTANCE** Deep-sea hydrothermal vents are dominated by several invertebrate species. The establishment of symbiosis has long been thought to be the key to successful colonization by these sedentary species in such harsh environments. However, the relationships between symbiotic bacteria and their hosts and their role in environmental adaptations generally remain unclear. In this paper, we show that the distribution of three host species showed characteristic niche partitioning in the Manus Basin, giving us the opportunity to understand how they adapt to their particular habitats. This study also revealed three novel genomes of symbionts from the snails of A. boucheti. Combined with a data set on other ectosymbiont and free-living bacteria, genome comparisons for the snail endosymbionts pointed to several genetic traits that may have contributed to the lifestyle shift of *Epsilonproteobacteria* into the epithelial cells. These findings could increase our understanding of invertebrate-endosymbiont relationships in deep-sea ecosystems.

## Abstract (precis)

Deep-sea hydrothermal vents are dominated by several invertebrate species. The establishment of symbiosis has long been thought to be the key to successful colonization by these sedentary species in such harsh environments. However, the relationships between symbiotic bacteria and their hosts and their role in environmental adaptations generally remain unclear. In this paper, we show that the distribution of three host species showed characteristic niche partitioning in the Manus Basin, giving us the opportunity to understand how they adapt to their particular habitats. This study also revealed three novel genomes of symbionts from the snails of A. boucheti. Combined with a data set on other ectosymbiont and free-living bacteria, genome comparisons for the snail endosymbionts pointed to several genetic traits that may have contributed to the lifestyle shift of *Epsilonproteobacteria* into the epithelial cells. These findings could increase our understanding of invertebrate-endosymbiont relationships in deep-sea ecosystems.

## INTRODUCTION

Deep-sea hydrothermal vents are considered one of the most extreme environments in the world, where temperatures and hydrogen sulfide and heavy metal concentrations can reach extraordinarily high levels (1, 2) and the pH gradient is steep (3). Among the common organisms living in hydrothermal vents, some of the most abundant are chemolithoautotrophic bacteria, which generate energy for carbon fixation via the oxidation of vent-derived, reduced, inorganic chemicals (4), but invertebrates also flourish in such environments. The vents in the Manus Basin are dominated by bathymodiolin mussels, alvinellid polychaetes, and galatheid crabs, as well as provannid snails that occupy separate, distinctive habitats (5, 6). The key to these metazoans’ success in thriving in this harsh environment is their ability to establish symbiosis with autotrophic bacteria to acquire nutrients from surrounding fluids (7, 8). Symbiosis is a common ecological phenomenon in vent-dwelling invertebrates for support of their survival in extreme environments. Most of the symbionts found in this environment are chemosynthetic bacteria that provide nutrients like organic carbon and nitrogen to their marine invertebrate hosts (9). Generally, interactions between symbiotic microbes and their hosts have played an important role in shaping the ecology and evolution of many marine animals. For example, microbial community compositions differ among different niches of the same vent due to within-site variations in temperature and fluid chemistry (10). Thus, symbiotic bacteria are viewed as being a determining factor governing the distribution of small-size deep-sea macrofauna, such as mussels, tubeworms, and snails (6).

On the basis of their total biomass, the mussels, tubeworms, and snails are the dominant invertebrate life forms dwelling in the hydrothermal vents of the Manus Basin. The majority of bathymodiolin mussels host either thiotrophic or methanotrophic *Gammaproteobacteria* in their gill bacteriocytes, but some species can host both types simultaneously (11). Two recent studies have also presented evidence that multiple thiotrophic symbiont strains can coexist in one host (12, 13).

The majority of known deep-sea siboglinid tubeworms acquire a single *Gammaproteobacteria* ribotype as an endosymbiont from the surrounding environment (14). Through an infection-like process, these symbionts invade the skin of tubeworms and colonize the cells of a specialized organ called a trophosome, located in the mesodermal tissue (14, 15). Genomes of endosymbionts from several large marine worms known as vestimentiferans—including the seep-dwelling *Escarpia* and *Lamellibrachia* and the vent-dwellers *Riftia*, *Ridgenia*, and *Tevnia*—have been sequenced. Those data show that the sequenced endosymbionts possess great metabolic flexibility concerning carbon fixation in energy-rich reducing habitats, and the intraspecific variation in their carbon and nitrogen metabolism may be crucial to the holobionts’ ability to adapt to their environment (15, 16).

Provannid gastropods of the genus *Alviniconcha* are among the dominant inhabitants that harbor endosymbionts in hydrothermal vents (17, 18). Their symbionts are located within vacuoles in the enlarged ctenidium, and different lineages of *Alviniconcha* are associated with *Gamma*- or *Epsilonproteobacteria* (6, 19, 20). The *Alviniconcha* gastropods are among the few species to have established endosymbiotic relationships with *Epsilonproteobacteria* (21), offering researchers the opportunity to elucidate characteristic genomic features of their endosymbionts.

Additionally, significant differences among the habitats of these dominant vent species have been discovered (22–24). For example, depleted oxygen and nearly millimolar concentrations of sulfide have been reported from many vent sites where Alvinella pompejana thrives (25–27). The decapod vent shrimp, Rimicaris exoculate, forms aggregations in the turbulent, mixing interface of hot fluids and seawater along chimney walls, where the oxygen concentration is apparently high (28). In more diffuse vent fluids, where mussels and other dominant taxa have been found, sulfide levels rarely exceed 100 μM, and the water temperature is usually below 30°C (27). Such niche partitioning in space can also influence microbial distributions in the deep-sea vent environment. Recently, the high divergence of the same-ribotype symbionts in different mussel species was reportedly related to geographical features (29). Earlier work showed how the distribution of *Alviniconcha* host-symbiont associations is influenced by geochemical gradients found in the Lau Basin (6), and a metatranscriptomic study revealed differences in symbiont metabolism related to environmental chemistry and symbiont phylogeny (30). However, studies that compare metabolic differences of symbionts from different habitats remain limited, and this knowledge gap hinders our understanding of how symbionts contribute to the environmental adaptations of deep-sea macrofauna.

This study explored the genomic characteristics and metabolic potential of symbionts of dominant invertebrate species in the Manus Basin (Papua New Guinea). Bathymodiolin mussels, siboglinid tubeworms, and provannid snails were chosen for genome sequencing and data mining to reveal their phylogeny and genomic features that may contribute to niche partitioning of macrofauna in the local ecosystem. Using more genomes from endosymbionts, our comparative analysis revealed adaptive symbiotic characteristics for *Epsilonproteobacteria*, as well as metabolic differences for symbionts in separate host groups that dwell in niches distinguished by their geochemical characteristics.

## RESULTS AND DISCUSSION

### Distribution of metazoans in vent field.

The distribution of macrofauna in the Manus Basin showed characteristic niche partitioning for different groups of coexisting animals. The plume fluid and the chimney-like “smoker” walls were inhabited by *Rimicaridinae* shrimps and *Alviniconcha* snails, respectively. A short distance away from the smoker, biomass was much lower and Munidopsis lauensis dominated. Further afield, near low-temperature fluid, large clusters of the tubeworm Arcovestia ivanovi were observed. On the edge of the vent field, Bathymodiolus manusensis dominated, with small patches of Munidopsis lauensis and *Alvinocaris* spp. At the vent site, provannid snails colonized the high-temperature black chimney walls and siboglinid tubeworms were found in the lower-temperature, diffuse fluids, while the bathymodiolin mussels occurred in the outer region of the vents (ca. ∼10 m away from the black smoker chimney).

### Phylogeny of host invertebrate species.

BLAST of cytochrome *c* oxidase subunit I (COI) sequences confirmed that the specimens used in this study belonged to Bathymodiolus manusensis (100.00% nucleotide identity to the B. manusensis mitochondrion partial genome with accession number KY270856), Arcovestia ivanovi (99.63% nucleotide identity to the A. ivanovi sequence with accession number AB073491), and Alviniconcha boucheti (99.37% nucleotide identity to the A. boucheti sequence with accession number KF467840).

### Endosymbiont composition and phylogeny.

**FIG 1:**
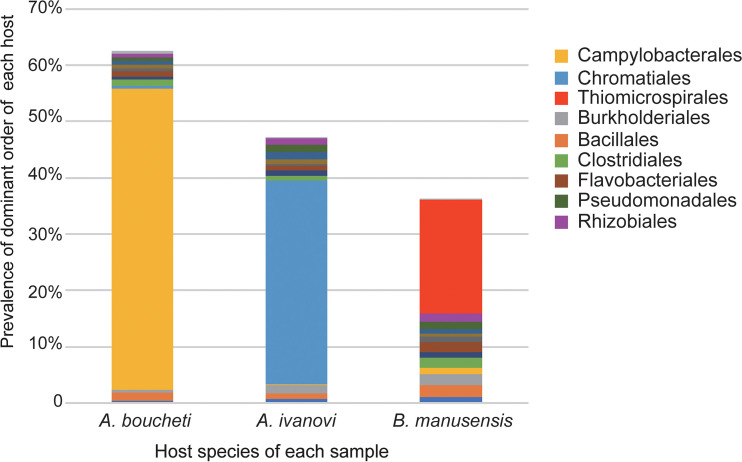
Reads classified and ranked by order in each sample. The top order in A. boucheti is *Campylobacterales*, from the class *Epsilonproteobacteria*. The top orders in A. ivanovi and B. manusensis, respectively, are *Chromatiales* and *Thiomicrospirales*, both from the class *Gammaproteobacteria*.

Reads obtained from each sample were classified to the order level by using Kraken2 (31). The top 10 bacterial orders dominating the microbiological communities in each sample are shown in Fig. 1. In gill samples of the snail A. boucheti, approximately 53.3% of the total reads belonged to *Campylobacterales* (*Epsilonproteobacteria*). In the trunk of the tubeworm A. ivanovi and gills of the mussel B. manusensis, the top-ranked orders were *Chromatiales* and *Thiomicrospirales* (*Gammaproteobacteria*), accounting for 36.0% and 20.2% of the total reads, respectively. Minor taxa in each sample might be environmental bacteria that were affiliated with the surface of the sampled tissue. In the A. boucheti muscle sample, the total read counts for top bacterial orders were much lower, with the top order being *Bacillales*, accounting for 3.32% of the total reads (data not shown).

One high-quality endosymbiont draft genome each was generated for B. manusensis and A. ivanovi, while three draft genomes were generated for A. boucheti. The endosymbiont assembly of B. manusensis showed high strain heterogeneity, as previously reported. Therefore, a representative genome containing contigs with high coverage similar to the single-copy gammaproteobacterial genes was extracted and used for the functional analysis (Note S1 in the supplemental material). Strain heterogeneity in symbionts of bathymodiolins has been reported for Bathymodiolus septemdierum and Bathymodiolus brooksi (13, 32), and this might facilitate rapid or novel adaptations to a changing environment. The general status of the genomes obtained and their corresponding quality assessments are provided in Table 1. The symbiont draft genomes obtained for each species were nearly complete (85.7% to 98.6%).

**TABLE 1:** Summary of the symbiont assemblies

Characteristic	Value for symbiont assembly from indicated host				
	Bathymodiolus manusensis: BAMAsym	Arcovestia ivanovi: ARCOsym	Alviniconcha boucheti		
			ALBOsym1	ALBOsym2	ALBOsym3
Genome size (bp)	2,490,315	4,260,951	2,257,243	1,527,333	1,405,570
No. of contigs	667	275	268	141	72
GC content (%)	38.9	54.1	37.3	38.8	40.0
*N*_50_ value	4,709	32,059	14,436	18,588	27,369
Completeness (%)	85.73	98.63	98.36	90.57	89.47
Contamination (%)	4.71	2.014	1.639	0.409	8.771
Lineage	*Gammaproteobacteria*	*Gammaproteobacteria*	*Epsilonproteobacteria*	*Epsilonproteobacteria*	*Epsilonproteobacteria*
No. of:					
Coding sequences	2,101	3,797	2,346	1,651	1,452
5S RNAs	1	1	0	0	0
16S RNAs	1	1	1	0	0
23S RNAs	1	1	0	0	0
tRNAs	33	45	39	30	34
GenBank accession no.	VCHC00000000	VCHD00000000	VCAW00000000	VCAX00000000	VCAY00000000

For 16S rRNA gene sequence-based analysis, a single 16S rRNA gene sequence was recovered from ARCOsym (symbiont genome from the tubeworm Arcovestia ivanovi) and ALBOsym1 (symbiont genome from the snail Alviniconcha boucheti) using BLAST search against the Silva database, and only one ribotype was available for each symbiont based on the single-nucleotide polymorphism (SNP) calling results. However, there was no candidate 16S rRNA gene included in the draft genomes of ALBOsym2 and ALBOsym3. BLASTing the metagenomic sequences against the Silva database did reveal two contigs containing 16S rRNA genes belonging to the same taxonomic groups as these draft genomes and with similar short-read mapping depths. Thus, these two sequences were included in the 16S rRNA gene-based phylogenetic constructions. For all the 16S rRNA genes included in draft genomes, no SNPs were detected, indicating a single-ribotype status of the symbionts.

**FIG 2:**
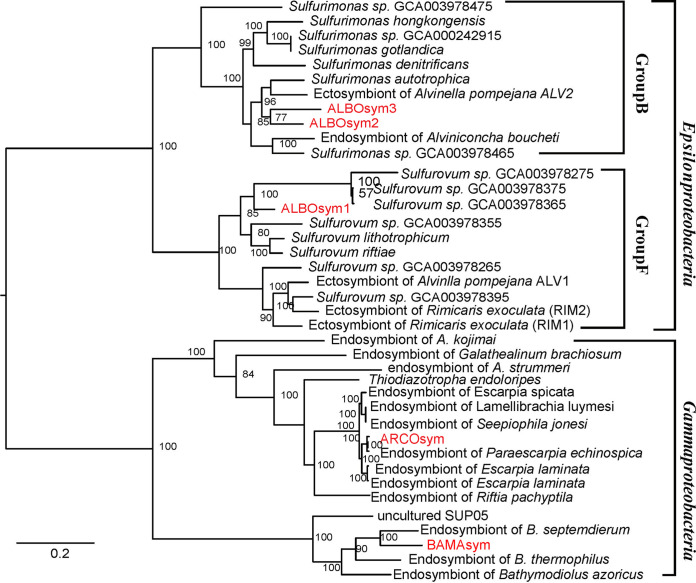
Phylogenomic tree based on single-copy-ortholog sequences showing the phylogenetic positioning of symbionts in *Proteobacteria*. Bootstrap values were calculated based on 100 replicates. Genomes marked in red were sequenced and assembled in this study.

A phylogenomic tree was constructed based on 11 single-copy orthologs (3,195 amino acids). The tree topology was consistent with that of a separately constructed phylogenomic tree of *Sulfurovum*, *Sulfurimonas*, and *Gammaproteobacteria* (15,441 amino acids [aa], 70,059 aa, and 68,731 aa, respectively) (data not shown). The 16S rRNA-based phylogenetic analysis and phylogenomic analysis provided solid and consistent results (Fig. 2; Fig. S1 and Note S2). The phylogenetic analysis robustly placed symbionts of B. manusensis in a clade with other SUP05-like thiotrophic endosymbionts of bathymodiolins. The symbiont of the siboglinid tubeworm *Arcovestia* was closely related to those of *Escarpia* sp. and *Lamellibrachia* sp. Two of the symbiont genomes obtained from A. boucheti, namely, ALBOsym2 and ALBOsym3, showed close affinity to *Sulfurimonas*, referred to as *Epsilonproteobacteria* group B, while the other genome, ALBOsym1, was clustered within *Epsilonproteobacteria* group F (13, 32, 33), being the main source of epibionts of deep-sea invertebrates (A. pompejana, Riftia pachyptila, and others) and free-living bacteria in deep-sea hydrothermal vents (19, 34).

It is known that *Alviniconcha* snails harbor diverse symbiont ribotypes within and among their populations (34). A dual-phylotype, gammaproteobacterial symbiont was observed in individual snails (6), but multiple phylotypes of epsilonproteobacterial symbionts within one host have not been reported previously. Interestingly, three genomes and three 16S rRNA sequences were extracted from the metagenome of A. boucheti symbionts, whereas previous research reported only single 16S phylotypes in snail individuals that host *Epsilonproteobacteria* (6). Currently, it is hard to draw any firm conclusions concerning symbiont diversity in A. boucheti snails, since only one snail sample was used for metagenomic sequencing in our study, and the multiple genomes obtained may also have included ectosymbionts from the snail gill tissue. Nevertheless, all 16S rRNA gene sequences recovered in this study had a high abundance in the metagenome sequences and displayed considerable similarity to known *Alviniconcha* endosymbionts (35). We thus propose the possible existence of a multisymbiosis relationship in A. boucheti, which has never been reported before. Additional investigation, in studies that include fluorescence *in situ* hybridization (FISH), scanning electron microscopy (SEM), or isotopic analysis, along with more biosamples, are needed to validate this finding and further understand the interactions among each of these symbionts and their A. boucheti hosts.

In addition, the symbiotic constraints between the bacteria and *Alviniconcha* seem less rigid than those in mussels and tubeworms. The primary endosymbionts of A. boucheti belong to different genera found dwelling at different sites (35), whereas this is unusual for mussels and tubeworms (36).

### General genomic profiles of the symbionts.

**FIG 3:**
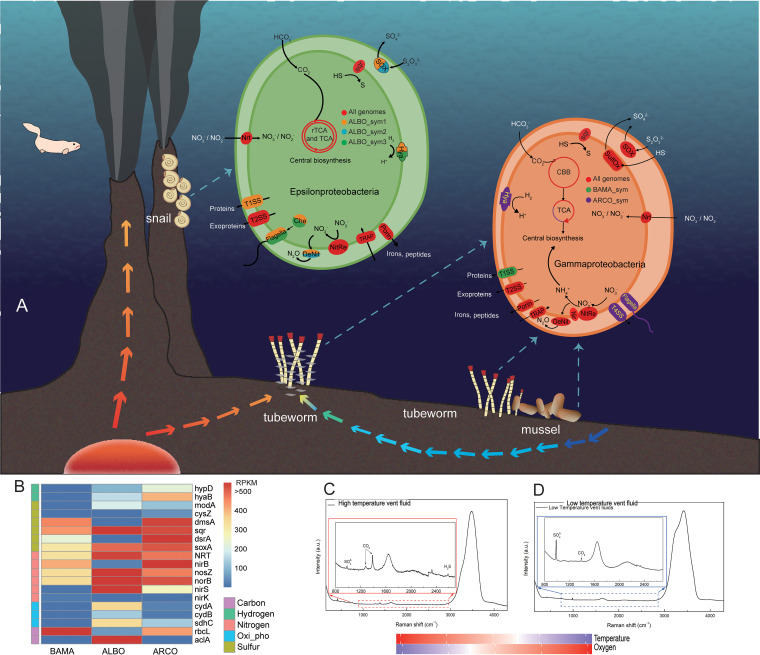
(A) Schematic view of the distribution of B. manusensis (BAMA), A. ivanovi (ARCO), and A. boucheti (ALBO) and potential metabolic pathways of their symbiotic *Gamma-/Epsilonproteobacteria* in the PACManus hydrothermal vent field. In *Epsilonproteobacteria*: red, pathways presented in all three genomes; orange, pathways found in ALBOsym1; blue, pathways found in ALBOsym2; green, pathways found in ALBOsym3. In *Gammaproteobacteria*: red, pathways found in both BAMAsym and ARCOsym; green, pathways found in BAMAsym; purple, pathways found in ARCOsym. (B) The relative abundances of key genes confirming the presence or absence of corresponding metabolic pathways. Proteins encoded by key genes are as follows: HypD, hydrogenase maturation factor; HyaB, hydrogenase-1 large subunit; ModA, molybdate-binding protein; CysZ, sulfate transporter; DmsA, dimethyl sulfoxide reductase; Sqr, sulfide-quinone reductase; DsrA, sulfite reductase dissimilatory-type subunit alpha; SoxA, l-cysteine *S*-thiosulfotransferase subunit; NRT, high-affinity nitrate transporter; NirB, nitrite reductase (NADH) large subunit; NosZ, nitrous-oxide reductase; NorB, nitric oxide reductase subunit B; NirS, nitrite reductase; NirK, nitrite reductase; CydA, cytochrome *bd* ubiquinol oxidase subunit 1; CydB, cytochrome *bd* ubiquinol oxidase subunit 2; SdhC, succinate dehydrogenase cytochrome subunit; RbcL, ribulose bisphosphate carboxylase large chain; AclA, ATP-citrate lyase alpha-subunit. RPKM, reads per kilobase per million. (C and D) Raman spectra of high-temperature vent fluids (C) and low-temperature vent fluids (D).

The core metabolic pathways of each genome were reconstructed based on PGAP and KEGG annotations, and they are summarized in Fig. 3. The relative abundances of key genes involved in carbon, nitrogen, hydrogen, and sulfur metabolism were compared for each sample (Fig. 3).

### (i) Sulfur metabolism.

Reduced sulfur compounds are the predominant electron donors found in deep-sea vents (10). Sulfate transporters and genes encoding the Sox enzyme systems were identified in all symbionts, suggesting that the oxidation of thiosulfate serves as an important energy source. In contrast, dissimilatory sulfate reduction/oxidation genes were not detected in the symbionts of the snail. Sqr genes, which are believed critical for growth under high-sulfide concentrations (37), were found in all symbionts, but they occurred at relatively higher abundances in the genomes of snail symbionts than in the genomes of symbionts from mussel and tubeworm.

### (ii) Nitrogen metabolism.

Genes for nitrate respiration (denitrification or dissimilatory nitrate reduction or both), which provides alternative electron receptors when the oxygen level is low (38), were complete in all assembled genomes except that of ALBOsym3. However, nitrite reductase was only available in a small portion of the mussel’s symbiont strains. The collaboration required to remove toxic nitrite may strengthen the interdependence between strains and could underlie the observed substantial heterogeneity in BAMAsym (symbiont genome from the mussel Bathymodiolus manusensis). NO is known to function in host innate immunity and may hamper symbiosis at high concentrations. The NO reductase (Nor) was available in all the symbionts whether or not the denitrification pathway was complete, and it might participate in NO detoxification (39).

### (iii) Carbon and energy metabolism.

Carbon was fixed through either the Calvin-Benson-Bassham (CBB) cycle in *Gammaproteobacteria* symbionts or the reductive tricarboxylic acid (rTCA) cycle in *Epsilonproteobacteria* symbionts. Ni/Fe-hydrogenase (HyaAB) was available in symbionts of A. ivanovi and A. boucheti, whose habitats were adjacent to vent-derived fluids containing hydrogen, but it was absent in symbionts of B. manusensis specimens. Hydrogen seems to contribute to chemosynthesis in the symbionts in tubeworms and snails, as reported for other vents’ invertebrates (6, 35). Cytochrome *cbb*_3_-type oxidases were present as the terminal oxidase in all symbionts. Cytochrome *cbb*_3_ oxidases possess high affinity for oxygen, which is essential under hypoxic conditions, and hence, it may be important in the adaptation of symbionts to microaerobic or anaerobic microenvironments inside bacteroids (40). Cytochrome complex *bd*, with its higher oxygen affinity (41), was encoded by the genome of the snail symbiont that dwells in the vent habitat with the lowest oxygen.

### (iv) Secretion system.

All five symbiont genomes contained genes encoding the type II secretion system (T2SS), which is conserved in Gram-negative bacteria and commonly found in bacterial pathogens of plants, animals, and humans (42). Proteins are first transported across the cytoplasmic membrane by the Sec or Tat pathway, then folded in the bacterial periplasm, and finally secreted into the extracellular environment by T2SS (43). The proteins exported by T2SS are diverse, including toxins, virulence factors, cytochromes, and a broad range of enzymes (44). The substrates transported by T2SS in these symbionts might thus be important for providing nutrients to the host, for helping symbionts to adjust to their environment, or to facilitate the establishment of symbiosis (45, 46). Furthermore, transporters with multiple functions were found to be abundant in the genomes of the symbionts, including, *inter alia*, those for lipids, amino acids, minerals, and polysaccharides.

### (v) Toxin-antitoxin.

There were numerous genes for toxins and antitoxins in all the symbionts examined. Under stressful conditions, the degradation of antitoxins can be triggered, leading to the activation of certain toxins and the induction of specific cellular functions. Toxin-antitoxin (TA) systems can also contribute to intracellular survival of invading bacteria during eukaryote interactions in both plants and animals by regulating growth arrest, adaptation, and cell death (47, 48).

### (vi) Glycosylation.

Genes necessary for glycosylation, especially those targeting the exocellular matrix, were one of the dominant families present in all symbionts sequenced in this study. Glycans are important ligands for extrinsic recognition, and they can mediate symbiont modulation of host immune responses (49). Both lipopolysaccharide (LPS) and glycoproteins can induce specific recognition of bacteria during the establishment of symbiosis and likewise aid the symbionts in host immune evasion (49). In the latter context, mutations to the polysaccharide locus in Vibrio fischeri conferred defects in their ability to colonize squid hosts (50).

### Protein family homolog identification and comparison.

Pfam-based comparison of the coding genes in each endosymbiont was carried out (Fisher’s exact test, *P* < 0.05) to reveal the putative genetic basis for the deep-sea vent habitat adaptations. Detailed information on this can be found in Table S3. Family expansion of efflux transporters—especially those of the outer membrane (outer membrane efflux protein [OEP]), chaperones (DnaJ), and the reactive oxygen species (ROS)-responding gene products (PqiA [thioredoxin])—was found in the snail A. boucheti endosymbionts, which would suggest their involvement in stress tolerance of high-level heavy metals and sulfide (51, 52). In addition, molybdopterin-binding domains, possibly involved in nitrate or sulfate respiration in response to low oxygen levels (53), and the integrase domain, involved in horizontal gene transfer, were also found to be expanded. In comparing ARCOsym and BAMAsym, the genomes of snail symbionts consisted of a smaller number of gene families related to the two-component system, host-bacteria interaction, virulence factor, and eukaryotic-like proteins. Virulence factors and eukaryotic-like proteins are known to contribute to symbiosis interactions (54). The contraction of such genes lends further support to looser symbiosis constraints in this snail species.

In the tubeworm A. ivanovi’s endosymbiont, the Na^+^/H^+^ ion antiporter, which may be involved in pH regulation, in addition to the sulfur globule protein, which may aid in sulfide detoxication by storing sulfur during the oxidization of reduced sulfur compounds (55), were both expanded. Many gene families linked to physiological responses to environments were found to be significantly expanded, including those for c-di-GMP regulation, sigma factors, chemotaxis, and two-component systems. The expanded gene families we discerned also contain several gene families participating in symbiosis interactions, including eukaryotic-like proteins like Ankyrin repeats and proteins involved in host attachment. The gene families that contracted were mainly related to virulence factors and the regulation of host-symbiont interactions.

In the B. manusensis endosymbiont genome BAMAsym, the gene families or domains related to attachment and host-symbiont interactions, virulence factors, posttranscriptional regulation, toxins, methylases, and eukaryotic-like proteins were all found to be expanded. In contrast, gene families involved in environmental sensing and response were contracted, including genes for two-component systems, chemotaxis, and c-di-GMP regulation.

### Niche adaptation revealed by comparative genomics.

The three metazoan species sampled in PACManus showed a distinct niche partitioning pattern, in that provannid snails colonized the high-temperature black chimney walls characterized by high-temperature sulfide and heavy metals but low oxygen levels and pH. Bathymodiolin mussels were located in the outer region of the vents, where the environment resembles seawater in having much lower concentrations of sulfide, protons, and heavy metals. Siboglinid tubeworms were found in the diffuse fluid habitats featuring intermediate conditions (56); however, dissolved concentrations of heavy metals are higher in diffuse fluid because of its lower concentration of sulfide (57). The *in situ* Raman observations and onboard measurements confirmed the low pH near chimney walls (∼5.0) and of diffusing fluid (∼6.5) compared with general seawater pH (∼7.5 to 8.4).

As discussed above, the high-oxygen-affinity *bd*-type cytochrome complex and more energy-efficient rTCA cycles (58) in the snail’s symbionts may support their survival under hypoxic conditions. Furthermore, the relatively high abundance of denitrification genes in the snail symbionts, as well as their tendency to utilize a soluble NADH-dependent fumarate reductase, which functions well in the microaerobic and anaerobic environments (59), are features that are all well suited to persisting in a low-oxygen environment.

**FIG 4:**
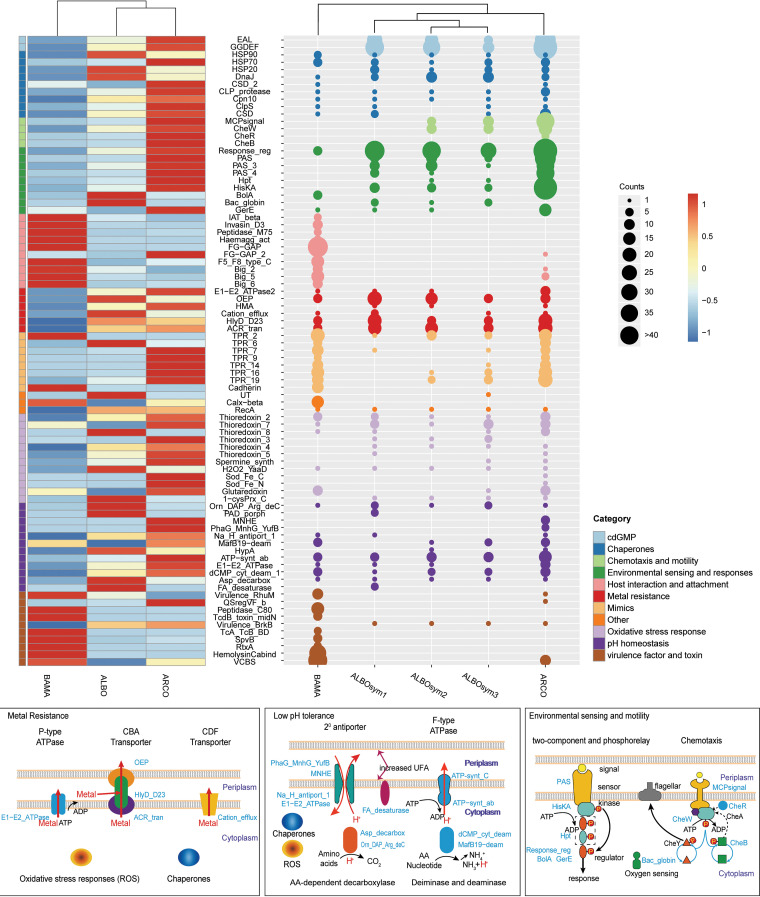
Gene family abundances and sizes conveyed by a heat map and gene number counts. Gene abundances calculated by read mapping to reference Pfam domains are presented in the heatmap on the left (RPKM was normalized by z-score). Gene family enrichment (indicated by size of dot) is shown in the count plot on the right. Gene family categories are shown in different colors. Schematic views of pathways related to metal resistance, low pH tolerance, and environmental sensing and motility are presented in the boxes at the bottom.

To elucidate the relationship between gene family size and its role in the adaptation of symbionts in each host, we then compared the numbers of key gene families in the sequenced genomes and confirmed their abundances by mapping reads to the reference Pfam domains (Fig. 4). Gene family abundances estimated by gene number counts and read mapping were consistent with each other, evincing the validity of our binned data set. In general, a number of gene families related to low-pH homeostasis, metal resistance, oxidative stress resistance, environmental sensing and responses, and chemotaxis and motility showed the highest abundances in the ARCOsym genome, followed by the ALBOsym genomes of symbionts of the vent-mouth dwelling snail, and yet, they had relatively low abundance in the BAMAsym genome of the symbiont of vent-periphery dwelling mussels.

### (i) pH homeostasis.

Genes related to pH homeostasis also showed higher abundance in the symbiont genomes from snail and tubeworm (Fig. 4). Correspondingly, low pH values (near chimney, ∼5.0, and diffusing fluid, ∼6.5) and high concentrations of heavy metals were detected in the habitats of snails and tubeworms by our *in situ* Raman observations and onboard measurements. In endosymbiont bacteria, protons can be expelled actively by proton-coupled ATPases and secondary active transporters (60). The latter may participate in proton regulation as the expanded P-type ATPases, which serve as proton antiporters and function to regulate pH homeostasis in symbionts of both snail and tubeworm species; in the latter’s symbiont, Na^+^/H^+^ antiporters were also found to be expanded. Nonetheless, proton levels can be reduced by either amino acid decarboxylases, which consume protons, or deiminases and deaminases, which generate ammonia (61); accordingly, more gene copies of pyridoxal-dependent decarboxylase and lysine decarboxylase were found to be present in the symbiont genomes of snails and tubeworms. Finally, modifying proton permeability through the cell membrane is another way to lessen proton permeability, namely, via the production of spermidine (62), and related genes were found to be expanded in symbionts of the tubeworm and snail.

### (ii) Metal resistance.

Genes related to metal transporters, including the P-type ATPase, CBA transporter, and CDF transporter, were globally expanded in the symbiont genomes from the snail and tubeworm (Fig. 4). Heavy metals can be eliminated by the above-mentioned energy-dependent ion efflux (63). In addition, the heavy metal-associated (HMA) domain, which is involved in the transport or detoxification of heavy metals in bacteria (64), was also abundant in the snail and tubeworm. To counterbalance possible protein damage caused by either low-pH or heavy metal stresses, the genomes of snail and tubeworm symbionts also contained high abundances of chaperone-coding genes, which function to maintain protein structures under abiotic stresses (65).

### (iii) Oxidative stress resistance.

Oxidative stress management is crucial for bacteria to survive in vent-driven environments because ROS can be generated in acidic, metal-/sulfide-rich conditions (66). As expected, genes associated with oxidative stress management, including thioredoxin, peroxide stress protein, and superoxide dismutase (SOD), were more abundant in symbiont genomes of the snail (ALBOsym) and tubeworm (ARCOsym) than in that of the mussel (BAMAsym). ROS degradation and oxidative stress management could also be complemented by protective mechanisms like the production of antioxidants (67), including spermidine and cobalamin, whose coding genes were also found to be abundant in snail and tubeworm symbionts. Under stressful, harsh conditions, aggregation and biofilm formation could also enhance the survival rate of bacteria (68). Extracellular polymeric substances (EPS) produced during biofilm formation can also protect free-living-stage symbionts by shielding them from the surrounding environment (69). Biofilm synthesis is often controlled by intracellular levels of the second messenger c-di-GMP, which is regulated by GGDEF- and EAL-domain-containing proteins (70). Genes encoding both domains were significantly expanded in all symbionts of the snail and tubeworm.

### (iv) Environmental sensing and responses.

Turbulence is another feature of habitats close to deep-sea vents. The strength of mixing between hot hydrothermal fluids and cold seawater is altered dramatically by the water currents and geological activities, giving rise to unstable thermal and chemical gradients (10). Since they are farther from vents, the habitats of the bathymodioline mussels are therefore much more stable regarding thermal and chemical gradients (71). Thus, effective responses to environmental changes are likely more pertinent for symbionts of the snails and tubeworms, especially for their free-living stages before symbiosis is established. The two-component system enables the symbionts to sense and respond to any changes in their surrounding environments by regulating metabolism, stress adaptation, and other perceptive processes (72). Known gene families related to this type of environmental sensing (e.g., HK and PAS) and their response regulators (RR) were all found to be expanded in the symbionts of near-smoker dwellers (ALBOsym and ARCOsym).

### (v) Chemotaxis and motility.

Motility-related genes could participate in the formation of symbiosis (73). As reviewed by Raina et al., motility and chemotaxis are widely involved in the active migration of symbiotic bacteria to a wide range of hosts (74) and deemed significant for the acquisition of environmental symbionts in deep-sea snails and tubeworms. Chemotaxis can further aid the symbionts of the squid to reach deep crypts within host tissues after successful infection (75). Similar strategies may apply during the migration of symbionts into the trophosome of the tubeworm. And yet, these genes were not detected in the symbiont of *Bathymodiolus* mussels, suggesting that it may use a different strategy to colonize host cells, one perhaps relying on an abundant virulence factor, toxins, or adhesins (76).

In contrast, the abundances of gene families classified as related to host interaction and attachment, virulence factors and toxins, and eukaryotic-like proteins were highest in the mussel’s symbiont and lowest in the snail’s. As mentioned above, a preponderance of gene families linked to host interaction and attachment, as well as virulence factors and toxins, might compensate for lost motility and function in the establishment of symbiosis. Meanwhile, eukaryotic-like proteins (ankyrin, TPR, and Eta) in pathogens and symbionts can reportedly alter host cellular processes to promote colonization and intracellular survival (54). A larger repertoire of the above-described genes is available in the B. manusensis symbiont, confirmed as expanded by Fisher’s exact test.

### Genome comparison of *A. boucheti* symbionts with related *Epsilonproteobacteria*.

The ability to establish endosymbiotic associations with marine invertebrates is more common in the *Alpha*-, *Beta*-, *Gamma*-, and Deltaproteobacteria (54, 77, 78). Although the members of groups F and B in *Epsilonproteobacteria* dominate the bacterial community in low-temperature habitats of hydrothermal vents, only limited examples of endosymbionts belonging to these taxa are available for analysis (34). The newly obtained draft genomes from the *Alviniconcha* gastropods were similar to those of endosymbionts discovered inside the gills of the host *Alviniconcha* sp. type II. The genomes obtained from the gastropods let us elucidate the possible adaptive genetic properties of the bacterial endosymbiotic lifestyle. In addition to this recently gathered data set, the genome of a known endosymbiont of A. boucheti (ALBOep) was also included.

**FIG 5:**
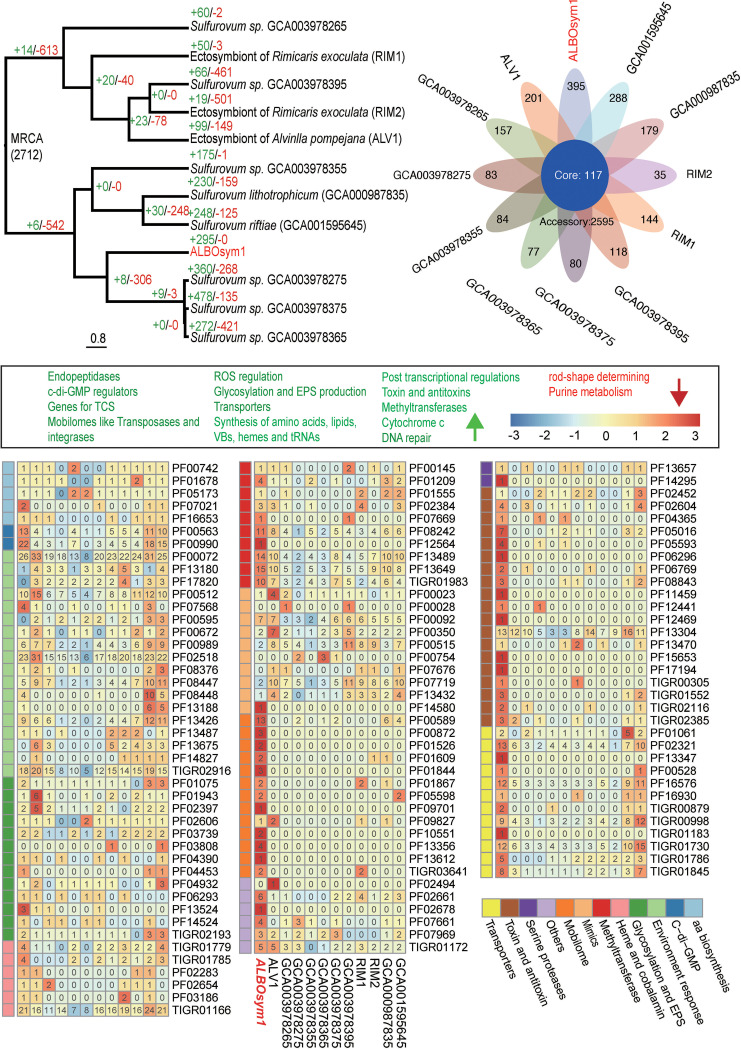
Genome comparison of ALBOsym1 with genomes of related species of genus *Sulfurovum*. Top left, gene family gains and losses along tree branches from the most recent common ancestor (MRCA) of *Sulfurovum*. “+” indicates the number of genes gained and “−” the number of genes lost. Top right, Venn diagram displaying numbers of core gene families, accessory gene families, and unique genes of ALBOsym1 and selected genomes of *Sulfurovum* obtained by Markov cluster algorithm (MCL) clustering. VBs, B vitamins. Bottom, heatmap display of relative gene abundances of selected Pfam families (based on Fisher test and Pfam enrichment) of ALBOsym1 and selected genomes of *Sulfurovum*. RPKM was normalized by z-score. Functional categories are indicated by different colors. The gene count of each Pfam domain is marked on the corresponding color block. Green arrow, list of expanded/orphan Pfam domains; red arrow, list of contracted/lost Pfam domains.

In the clade *Sulfurovum*, 295 gene families were found to be expanded compared to their abundances in its most recent ancestor (Fig. 5; Table S4), with 395 orphan genes identified. These genes were involved in numerous processes, including c-di-GMP regulation, two-component systems of inorganic nutrients, oxidative homeostasis maintenance, transporters, methylation, transposases, toxin-antitoxin systems, amino acid, tRNA, and vitamin B_12_ biosynthesis, endopeptidase, DNA repair and glycosylation, and the production of polysaccharides and lipoprotein. No contracted gene families were found. In ALBOsym1, 10 genes which may participate in the metabolism of purine and phospholipids were not found in the genome. Another gene, annotated as the rod shape-determining protein MreC, was also not detected.

**FIG 6:**
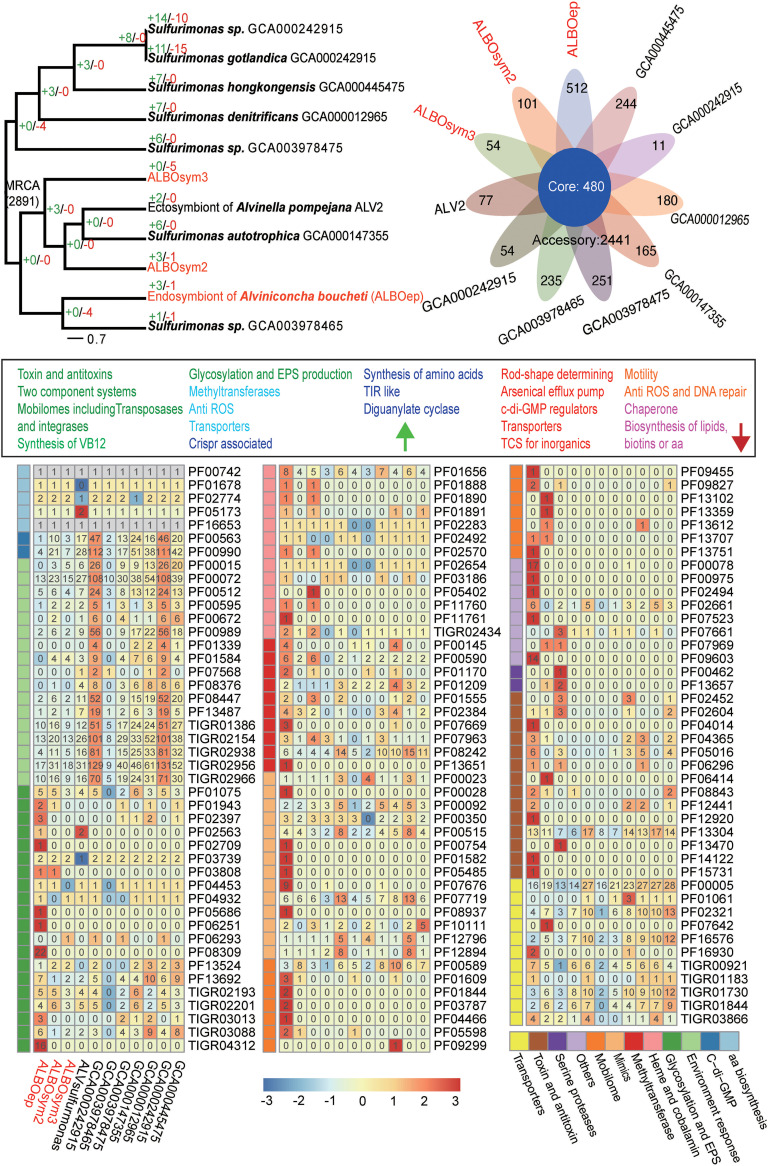
Genome comparison of ALBOsym2 and ALBOsym3 with genomes of related species of genus *Sulfurimonas*. Top left, genefamily gains and losses along tree branches from the MRCA of *Sulfurimonas*. “+” indicates the number of genes gained and “−” the number of genes lost. Top right, Venn diagram displaying numbers of core gene families, accessory gene families, and unique genes of ALBOsym2, ALBOsym3, ALBOep, and selected genomes of *Sulfurimonas* obtained by MCL clustering. VB12, vitamin B_12_; Crispr, clustered regularly interspaced short palindromic repeat. Bottom, heatmap display of relative gene abundances of selected Pfam families (based on Fisher test and Pfam enrichment) of ALBOsym and selected genomes of *Sulfurovum*. RPKM was normalized by z-score. Functional categories are indicated by different colors. Gene count of each Pfam domain is marked on the corresponding color block. Green arrow, list of expanded/orphan Pfam domains (green, found in ALBOsym2, ALBOsym3 and ALBOep; blue, found in two of the three above-mentioned genomes; indigo, found in one of the above-mentioned 3 genomes); red arrow, list of contracted/lost Pfam domains (red, found in ALBOsym 2, ALBOsym3, and ALBOep; blue, found in two of the three above-mentioned genomes; violet, found in one of the above-mentioned 3 genomes).

In the clade *Sulfurimonas*, there were 13, 6, and 2 gene families, respectively, expanded in ALBOep, ALBOsym2, and ALBOsym3, compared to their presence in their most recent ancestors (Fig. 6; Table S5). Meanwhile, totals of 512, 101, and 54 orphan genes were found in ALBOep, ALBOsym2, and ALBOsym3, respectively. These were involved in NADH oxidoreductase, toxin and antitoxin systems, mobilomes, vitamin B_12_ biosynthesis, glycosylation (ALBOep, ALBOsym2, and ALBOsym3), ROS management (ALBOsym2 and ALBOsym3), transporters (ALBOep and ALBOsym2), posttranscriptional modification (ALBOep), and synthesis of amino acids (ALBOsym2) and diguanylate cyclase (ALBOsym2). In terms of contraction, this happened to 1, 3, and 3 gene families compared to their presence in the most recent ancestors, as well as 41, 55, and 60 gene families specifically lost in the genomes of ALBOep, ALBOsym2, and ALBOsym3, respectively. These genes are known to participate in the maintenance of bacterial rod shape or be involved in arsenical efflux pump, c-di-GMP regulation, two-component systems on inorganic nutrients, transporters (ALBOep, ALBOsym2, and ALBOsym3), chemotaxis and motility (ALBOsym2 and ALBOsym3), ROS management (ALBOsym2 and ALBOsym3) and DNA repair (ALBOsym2 and ALBOsym3), chaperones (ALBOsym2), and the biosynthesis of lipids (ALBOsym2), biotins (ALBOsym2), and amino acids (ALBOsym3). The Pfam/TIGR expansion and contraction analysis of symbiotic gene families compared to those of the nonsymbiotic backgrounds support our results from the ortholog-based analysis mentioned above. Notably, the tetratricopeptide repeats were significantly contracted in ALBOsym1.

Nutrient interdependence strengthens the interactions between host and symbionts, leading to strong and stable symbiosis (79, 80). Transporters for carbohydrates, lipids, and amino acids were all available in the symbionts’ genomes, and they were even expanded in some symbionts. The carbon fixed via chemoautotrophy is generally utilized for host nutrition, according to a recent isotopic study (81). We also found that genes related to the biosynthesis of vitamin B_12_ appeared in the orphan-/expanded-gene list of all endosymbionts in the snail. This vitamin cannot be *de novo* synthesized by the host. Hence, the capacity of endosymbionts to synthesize vitamin B_12_ should be important in establishing symbiont-host relationships in A. boucheti. The importance of vitamin B_12_ in symbiotic associations has already been reported for algae and insects (82, 83). In contrast, the key genes necessary for purine/biotin biosynthesis were absent in some strains we examined, so they may acquire purine or intermediate forms from the host. Beyond providing nutrition to a host, symbionts can contribute to its protection against pathogens as well (84).

Compared with their presence in the nonendosymbiont species, gene families of TA systems were either expanded or specifically gained in all the endosymbionts of *Epsilonproteobacteria*. Notably, a type II antitoxin was only available to the endosymbionts, highlighting its necessity in the interaction of symbiosis. The TA system is crucial in mediating various cellular processes and stress-related adaptations, and it is believed to play multiple, critical roles in enhancing host-symbiont interactions (84, 85). According to one study of *Rhizobium*, a mutation to the gene encoding an antitoxin can lead to repressed symbiosis (86). TA systems are increasingly reported in more symbionts as functioning as molecular weapons to allow survival inside the host cell (86–88).

Methylation is another vital regulatory mechanism that can operate during symbiosis establishment in facultative endosymbionts like Regiella insecticola (89). Methylations around the symbiosis island have been shown to drive the differential expression of genes involved in all phases of symbiotic development (90). Since methylation-related genes were found in gene categories of both the expanded and orphan gene families, we propose that methylation may figure prominently in the symbiosis between *Epsilonproteobacteria* and the snail. As to the important roles of glycosylated protein and lipids, these was discussed above. Their involvement in symbiosis is also supported in *Epsilonproteobacteria* endosymbionts, especially for the primary symbionts (i.e., those symbionts highest in abundance) of the snail, by the evidence for expansion of glycotransferase- and LPS biosynthesis-related gene families.

Unlike the many *Sulfurimonas* and *Sulfurovum* species having a curved-rod shape, the symbionts found in the snail are egg-like or spherical in form (91). Interestingly, the ortholog annotated as the rod shape-determining protein MreC was lost in the genomes of two symbionts in A. boucheti. Depletion of MreCD in rod-shaped bacteria leads to the formation of spherical cells (92). We therefore hypothesize that a spherical shape is the result of a putative selection pressure for life stages inside the host cells.

c-di-GMP is the most common and important second-messenger molecule used to regulate physiological processes in bacteria, including their biofilm formation, life-history transition, motility, and virulence (93). c-di-GMP controls the ability of bacteria to interact with their biotic environment, including other bacteria and eukaryotic cells, and typically occurs at high concentrations during the initiation of symbiosis (93, 94). Levels of c-di-GMP may be regulated positively by genes encoding proteins with a GGDEF domain and negatively by genes encoding proteins with an EAL domain (70). However, the evolutionary fate of the c-di-GMP-regulating families differs between members of *Sulfurimonas* and *Sulfurovum*. Genes encoding proteins containing both GGDEF and EAL domains were expanded in the endosymbionts in *Sulfurovum* but contracted in *Sulfurimonas*, and yet all are adaptive to the endosymbiotic life mode. The respective changes in the sizes of gene families can function to influence both physiological and metabolic properties (95). More work, however, is needed to understand the effects of the changing diversities of GGDEF and EAL genes in the endosymbionts of A. boucheti.

### Conclusion.

The importance of endosymbionts in deep-sea metazoans is without question. However, the detailed role they play in assisting the host in environmental adaptation remains unclear. In this study, we explored the endosymbiont genomes of B. manusensis, A. ivanovi, and A. boucheti, each occupying a distinct niche at the PACManus hydrothermal vent. Genome comparisons revealed that the abundance of certain gene families (such as those involved in low-pH homeostasis, metal resistance, oxidative stress resistance, etc.) in each endosymbiont genome was consistent with the geochemical characteristics of their habitat. In addition, genome comparisons of A. boucheti endosymbiont genomes with other related genomes from *Epsilonproteobacteria* also illustrated certain traits that might be important in symbiosis establishment by members of *Epsilonproteobacteria*. Our work demonstrated the potential adaptive features in deep-sea endosymbionts using genome comparison analysis. However, some questions remain. For instance, the multisymbiosis of A. boucheti needs to be confirmed in the future, using more biosamples and methods such as FISH or SEM. Moreover, whether or under which conditions particular genes are expressed was not explored in the current study; future transcriptomics or proteinomics work should provide more insight into this question.

## MATERIALS AND METHODS

### Ethics statement.

All the specimens were used in the experiments in accordance with the guidelines and regulations issued by the State Council of the People’s Republic of China (Chinese Regulation on the Administration of Laboratory Animals, 2017 revision, http://www.gov.cn/gongbao/content/2017/content_5219148.htm).

### Sample collection, environmental observations, DNA extraction, and sequencing.

**FIG 7:**
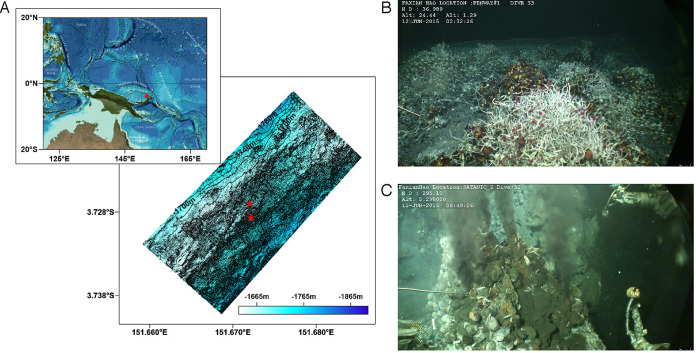
Location and sampling site images of the PACManbus hydrothermal vent area. (A) Regional location map and microbathymetry of PACManus. The top image was reproduced from the GEBCO world map 2014 (www.gebco.net), and the bottom image was created using Surfer program version 8.0 with processed bathymetric grid data (5-m by 5-m cells). (B) Bathymodiolin mussels and tubeworms observed in the low-temperature hydrothermal vent fluids. (C) Provannid snails on the surface of a black chimney.

The specimens used in this study, including three bathymodiolin mussels, a cluster of siboglinid tubeworms, and one provannid snail, were all collected from the Manus Basin by the scientific vessel *KEXUE* in June 2015 using either a television-controlled capture device or the remotely operated vehicle (ROV) *Faxian*. Meanwhile, *in situ* environmental parameters of high-temperature and low-temperature vent fluids were documented by using a Raman insertion probe (96) and a thermometer chain (TJ200) immediately before samples were taken. General information about the sample collection sites can be found in Fig. 7 and Table 2. Immediately after obtaining them, all live specimens were first dissected on board the ship and then preserved at −80°C immediately after their dissection.

**TABLE 2:** Locations and characteristics of the deep-sea vent sampling sites

Site	Specimen type(s)	Latitude	Longitude	CO_2_ (mmol/kg)^*a*^	H_2_S (mmol/kg)^*a*^	pH^*b*^	Temp (°C)
PAC-Satanic	Provannid snails	3°43′36.981″S	151°40′18.92″E	167.4	5.41	4.7	106
Fenway	Tubeworms and bathymodiolin mussels	3°43′42.084″S	151°40′21.088″E			6.5	21

a The concentrations of CO_2_ and H_2_S were calculated based on *in situ*-measured Raman spectra.

b pH was calculated based on Raman data and measured on board the vessel.

Symbiotic bacterial DNA was extracted from the gills of mussels, the trophosome of tubeworms, and ctenidium tissue of snails using a previously described methodology, albeit with minor modifications (97). Briefly, the dissected tissues from each individual animal were homogenized with sterile phosphate-buffered saline (PBS) buffer (supplemented with 10-fold diluted EDTA and 2% NaCl). The homogenate was centrifuged at 400 × *g* for 5 min, and the supernatant filtered sequentially through 10-μm, 5-μm, and 3-μm Millipore nitrocellulose membranes to remove any cell debris. Finally, the filtered liquids were centrifuged at 8,000 rpm for 5 min to obtain bacterial pellets. Bacterial genomic DNA was extracted with an EZNA D3350 bacterial DNA kit (Omega Bio-Tek, Norcross, GA, USA).

To facilitate the symbiont genome binning (metagenomics), host snail genomic DNA for genomic sequencing was prepared from pure muscle tissue by using an EZNA mollusc DNA kit (Omega Bio-Tek). Sequencing of genomic DNA was performed by Novogene (Novogene, Tianjin, China), using the Illumina 2 × 150 paired-end protocols on an Illumina HiSeq 4000 platform. Summaries of the reads generated for each sample can be found in Table S1 in the supplemental material.

### Symbiont composition analysis.

To show the composition of the symbionts in each sample, quality-filtered sequencing reads were subjected to taxonomic classification by the *k*-mer-based lowest-common-ancestor (LCA) approach implemented in the Kraken2 (31) taxonomic sequence classification system using the NCBI nonredundant protein database.

### Genome assembly and functional annotation.

**FIG 8:**
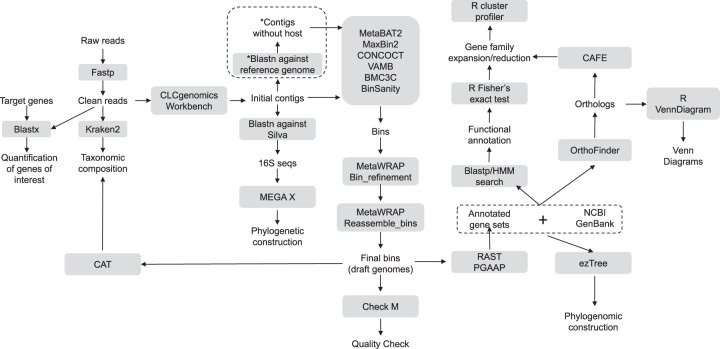
Schematic overview of metagenomic analysis performed in this study. Boxes in gray show the method or tools used in the corresponding analysis. *, only reads generated from B. manusensis were filtered using B. platifrons as a reference genome.

A schematic overview of the data analysis is shown in Fig. 8. The initial *de novo* assembly was carried out using CLC Genomics Workbench version 11.0 (Qiagen, Aarhus, Denmark), under its default configurations. For the *Bathymodiolus* samples, the host-derived contigs were filtered by a BLASTn search against the genome of Bathymodiolus platifrons. For all samples, short genomic assemblies (<1,000 bp) that could have biased the subsequent analysis were first excluded. Genomes were then binned based on their tetranucleotide frequency, differential coverage, and GC content, as well as codon usage, using 6 different binning tools: MetaBAT2, MaxBin2, CONCOCT, VAMB, BMC3C, and BinSanity (98–102). The binning results were refined using the MetaWRAP (version 1.2.1) package based on the bin quality assessment (completeness, >70, and contamination, <20) of different binners from CheckM (103, 104). Next, the selected bins for each deep-sea invertebrate sample were reassembled by using metaSPAdes and applying the MetaWRAP pipeline (103, 105). The results having the best quality were chosen for further analysis. Taxonomic classification of each bin was determined by CheckM and CAT (103, 106). The endosymbiont genome of B. manusensis was highly heterogeneous, indicating the possibility that its symbiotic bacteria were actually composed of multiple strains. Accordingly, the assembly of this genome was manually adjusted, as described in Note S1. The final metagenome-assembled genomes (MAGs) were annotated with both the Rapid Annotation using Subsystems Technology (RAST) server and the NCBI Prokaryotic Genome Annotation Pipeline (PGAP; https://www.ncbi.nlm.nih.gov/genomes/static/Pipeline.html) (107). All the genes were also searched for against the KEGG prokaryote database (108) and the Protein Families (Pfam) Database (109), using a threshold E value of 1e−6. For those genes not found in the KEGG pathways, a tBLASTn search of the corresponding candidate KEGG orthology proteins against the MAGs was performed to verify their absence.

### Relative gene abundance analysis.

Pfam domains or KEGG orthologs of interest were downloaded from their corresponding databases. Then, the sequenced reads were mapped to selected references using the Diamond BLASTx tool (version 0.9.21.122; default parameters with E value of 1e−6) (110). The read hit counts were first summarized and then normalized by the total count of clean reads to reads per million (RPM) to represent gene abundance through a homebuilt pipeline (108, 110, 111).

### Phylogenetic analysis.

PCR amplification and sequencing of the partial mitochondrial cytochrome *c* oxidase subunit I (COI) gene was carried out to validate the taxon of each host species, as described previously (18, 112). The COI sequences obtained were compared to reference sequences in the NCBI database by using BLAST searches.

The phylogenetic positioning of symbionts was analyzed based on both 16S rRNA gene (Note S2) and whole-genome sequences. Single-copy-ortholog selection and phylogenetic reconstructions were performed automatically using ezTree (113). Briefly, the predicted peptides were searched against the collection of known protein families (in Pfam and TIGR) by using hidden Markov models (HMM) (114) for the gene family assignment and single-copy-ortholog (SOG) extraction. Extracted SOGs were aligned using the PRANK (iterations = 50) tool and trimmed with trimal before performing the phylogenetic analysis using RAxML under the PROTGTR+CAT model (113–117). The robustness of the ensuing evolutionary tree was determined based on 100 bootstrap replicates (118). The reference genome sequences and selected orthologs used in this phylogenomic analysis are listed in Table S2.

### Comparative genome analysis.

Ortholog groups were determined by using OrthoFinder (version 2.2.3) with its default parameter settings (119). Homolog groups were divided into core, accessory, and unique gene sets. The core gene set comprised shared genes in all genomes, while the accessory gene set contained genes shared by at least two but not all genomes. The distributions of core, accessory, and unique genes were depicted using the R platform (version 3.5.1, VennDiagram package) (120). The expansion and contraction of gene families during the evolution of *Sulfurimonas* and *Sulfurovum* were reconstructed in CAFE (version 3.1) (121), using a *P* value cutoff of 0.05, by comparing the size of each gene family in the corresponding recent ancestor to the current node in the tree. Finally, to compare the size distributions of known protein families, the Pfam annotations were carried out with HMM. Gene family expansion/contraction events in the endosymbionts were identified using Fisher’s exact test (two tailed).

### Data availability.

The metagenome raw sequencing reads and assembled draft genomes were submitted to NCBI under BioProject accession numbers PRJNA511792 and PRJNA532304, respectively. The nucleotide sequences of cytochrome *c* oxidase subunit I for B. manusensis, A. boucheti, and A. ivanovi were submitted to GenBank under accession numbers MN106257, MK252698, and MK252699, respectively. The sequences of BAMAsym, ARCOsym, ALBOsym1, ALBOsym2, and ALBOsym3 were submitted to GenBank under accession numbers VCHC00000000, VCHD00000000, VCAW00000000, VCAX00000000, and VCAY00000000, respectively.

## Supplementary Material

Supplemental file 1

Supplemental file 2
