# Supplementary material for: Endosymbionts of Metazoans Dwelling in the PACManus Hydrothermal Vent: Diversity and Potential Adaptive Features Revealed by Genome Analysis
Source: Appl Environ Microbiol. 2020 Oct 15;86(21):e00815-20. doi: 10.1128/AEM.00815-20 (PMC7580541; doi:10.1128/AEM.00815-20)
Supplement: Supplemental file 1 [file AEM.00815-20-s0001.pdf]

Supplemental Information for:

Endosymbionts of vent-dwelling metazoans in PACManus —diversity and potential adaptive features revealed by genome analysis

Leilei Li, Minxiao Wang, Lifeng Li, Zengfeng Du, Yan Sun, Xiaocheng Wang, Xin Zhang, Chaolun Li

Supplementary note S1: Endosymbiont assembly of *B. manusensis*

It has been reported before that the symbionts of *Bathymodiolus* species constitute multiple subpopulations (1, 2). Similarly, our metawrap binning resulted in a draft genome with redundant copies of many conserved single-copy orthologic genes in *gammaproteobacteria*. The redundancy was raised due to the presence of highly similar gene blocks (HSG, >98% nucleotide similarity) in different contigs. Such high strain heterogeneity was not surprising as it has been found in the endosymbionts of *Bathymodiolus septemdierum* and *Bathymodiolus brooksi* (2, 3). For contigs with the same HSGs, the short-reads mapping showed that the depth for some contigs was much lower than others. When no obvious conflict HSGs were present in different contigs, only contigs with highest depth were retained. Since genes that are strain-specific usually have lower coverage than the conserved genes, we defined contigs with high coverage and are similar to single-copy gammaproteobacterial genes as CORE set (1).Thus, the final representative genome of 2.49Mbp in length was obtained.

Supplementary note S2: Phylogenetic analysis

Consistent with results from previous studies, *B. manusensis* hosted a single ribotype endosymbiont. In all 3 individuals, no SNPs in 16S rRNA genes were detected. The 16S rRNA gene sequence of BAMA_sym showed the highest nucleotide identity (98.63% identity, 100% coverage) to *B. thermophilus* thioautotrophic gill symbiont. The 16S sequence of ARCO_sym was the most similar to symbionts of *Lamellibrachia satsuma* with 99.48% nucleotide identity. At last, the 16S sequence of ALBO_sym1 showed 99.46% nucleotide identity to that of an uncultured bacterium of *Sulfurovum*.

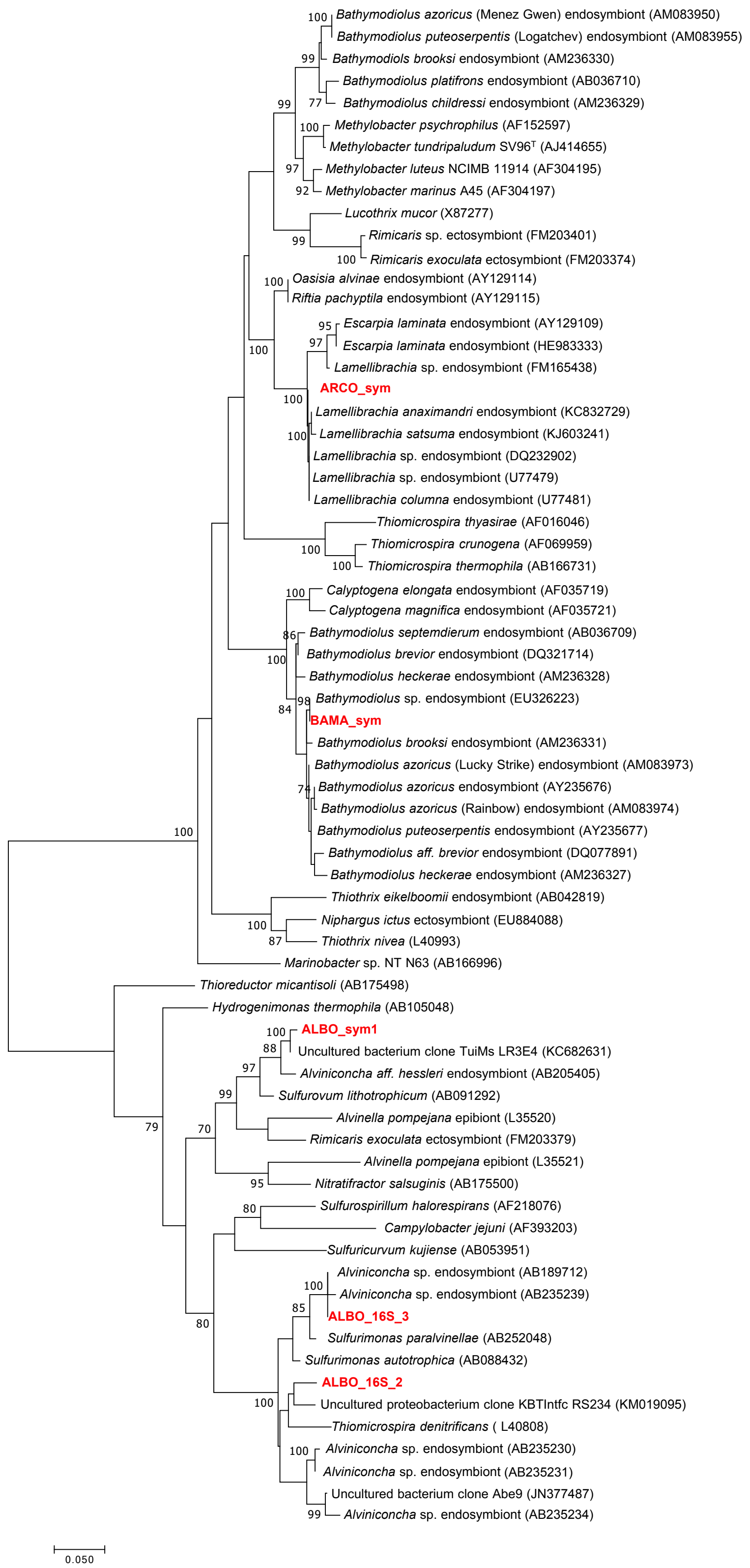

Supplementary Figure S1. Maximum likelihood tree based on nearly complete 16S rRNA gene sequences (1121nt) showing the phylogenetic positions of symbionts of *B. manusensis*, *A. ivanovi* and *A. boucheti*. The robustness of the branching is indicated by bootstrap values calculated for 1000 subsets.

References

1. **Ansorge R**, **Romano S**, **Sayavedra L**, **Kupczok A**, **Tegetmeyer HE**, **Dubilier N**, **Petersen J**. 2019. Diversity matters: deep-sea mussels harbor multiple symbiont strains. bioRxiv 531459.

2. **Ikuta T**, **Takaki Y**, **Nagai Y**, **Shimamura S**, **Tsuda M**, **Kawagucci S**, **Aoki Y**, **Inoue K**, **Teruya M**, **Satou K**, **Teruya K**, **Shimoji M**, **Tamotsu H**, **Hirano T**, **Maruyama T**, **Yoshida T**. 2015. Heterogeneous composition of key metabolic gene clusters in a vent mussel symbiont population. ISME J **10**:1–12.

3. **Romero Picazo D**, **Dagan T**, **Ansorge R**, **Petersen JM**, **Dubilier N**, **Kupczok A**. 2019. Horizontally transmitted symbiont populations in deep-sea mussels are genetically isolated. ISME J **110**:3229.
